# Cost-effectiveness of paediatric rapid genomic testing: a commentary

**DOI:** 10.12968/ijap.2025.0007

**Published:** 2026-01-02

**Authors:** W Weston, H Garrett, J Spencer, L Filipe, V Benedetto

**Affiliations:** Alder Hey Children’s NHS Foundation Trust; National Institute for Health and Care Research Applied Research Collaboration North West Coast; University of Sheffield; Lancaster University; University of Lancashire

**Keywords:** Genetic disorders, paediatrics, genomic medicine, rapid genomic testing, cost-effectiveness, commentary

## Abstract

Genetic disorders affecting children can lead to complex clinical pathways, fast clinical deterioration and infant mortality. Rapid genomic testing (RGT) can provide an early diagnosis and trigger appropriate clinical trajectories for children, ultimately improving health outcomes while potentially reducing costs.

In this commentary we critically appraised an existing economic evaluation of different RGT strategies which was set in an Australian hospital care setting. We highlighted how the authors suitably set the decision problem, perspective, model structure, costs and interpretation of the results of the economic evaluation. However, limitations associated with the short-term horizon employed and the lack of clinical and quality-of-life outcomes emerged. Building on the economic evaluation’s limitations, we formulated implications for practice reflecting recent developments on RGT and suggested avenues for future research. Addressing these limitations would further strengthen the economic case for RGT, provided that barriers and facilitation of its wide-scale implementation are taken into account. Ensuring wide-scale accessibility, effective systemic coordination and communication, offering education training opportunities to practitioners, reaping economies of scale to exploit likely changes in costs and differential cost-effectiveness of RGT for specific disorders and severity levels could be pursued in order for RGT to become part of the paediatric diagnostic toolbox.

## Introduction

Worldwide approximately 350 million people suffer from a rare disease, of which about 80% have genetic origin (Bick et al. 2019), with about half of these being children (Bick et al. 2019). Genetic disorders largely contribute to children’s mortality and admissions to intensive care, with clinical pathways for undiagnosed children often being complex (McDermott et al. 2022).

Genomic medicine can exploit cutting-edge technologies to predict and diagnose inherited and acquired disease and to personalise treatments (NHS England 2024). Rapid genomic testing (RGT) can prompt diagnosis of genetic disorders and appropriate clinical management (McDermott et al. 2022). The relative high cost of RGT can be compensated by reducing length of stay in critical care unit and in hospital overall (Stark and Ellard 2022; Xi et al. 2023).

As the clinical and cost-effectiveness of RGT gains promise, new evidence seems to justify its widespread adoption, as in the study by Goranitis et al. which aimed to evaluate the cost-effectiveness of rapid and ultra-RGT against standard genomic testing (SGT) within an Australian hospital setting (Goranitis et al. 2022). This commentary aimed to critically appraise that study (Goranitis et al. 2022) and expand upon the findings in the context of clinical practice.

## Overview of Goranitis et al.’s economic evaluation (Goranitis et al. 2022)

The economic evaluation by Goranitis et al. (Goranitis et al. 2022) compared four genomic testing strategies against SGT for the diagnosis of 36 infants and children with suspected monogenic disorders admitted to Royal Children’s and Monash Health hospitals In Melbourne. The strategies were: (1) RGT (initiated on day 13 from hospital admission with a 27-day results turnaround time); (2) RGT with early initiation, i.e. early RGT (initiated on day 2 with a 27-day turnaround); (3) ultra-RGT (initiated on day 13 with a 3-day turnaround); (4) ultra-RGT with early initiation, i.e. early ultra-RGT (initiated on day 2 with a 3-day turnaround). Adopting the perspective of the Australian healthcare system, the authors conducted three separate cost-benefit analyses with a time horizon coinciding with hospital stay (which lasted an average of 66 days with SGT).

In all the analyses, early ultra-RGT produced the highest mean per-child cost savings (compared to the other strategies). Savings were driven by the reduction in length of stay. On average across the three analyses, the total annual cost savings for the Australian healthcare system would be approximately AU$ 7.3 million, should early ultra-RGT be implemented. Considering an aggregate welfare gain of AU$ 3.3 million, the net annual benefit of early ultra-RGT would amount to AU$ 10.6 million approximately.

## Commentary

### Critical appraisal

We assessed the study by Goranitis et al. (Goranitis et al. 2022) using selected questions from quality appraisal tools (Critical Appraisal Skills Programme 2018; Drummond et al. 2015; Philips et al. 2004). While the authors appropriately determined the decision problem, perspective, model structure and costs of the economic evaluation and suitably interpreted the results, shortcomings on other aspects emerged.

The effectiveness of the RGT strategies was based on few clinical studies but was not substantiated by a systematic review. While the four proposed RGT strategies were detailed, the main comparator (SGT) was not sufficiently described. The selection of outcomes reflected the short time horizon of the analysis, focusing on the effects of the alternative strategies on the length of stay and number and type of investigations and treatments undertaken. However, the raw numbers for each strategy (e.g. length of stay) were not presented. Longer-term clinical and quality-of-life outcomes were not considered and could have illuminated the long-term value of RGT (Gyngell et al. 2019). The welfare gain analysis was accompanied by details on the measurement and valuation of the utilities preferences (Goranitis et al. 2021), but it was unclear how the aggregate welfare gain of AU$ 3.3 million was estimated. Probabilistic sensitivity analyses were mentioned for Analyses 2 and 3 but these results were not presented.

To enhance the generalisability of the results, the authors adopted two decision-analytic models (Analyses 2 and 3), but the underlying mean parameters’ values came from one clinical study only (Stark et al. 2018). Moreover, the heterogeneity in the type and severity of conditions of the children population was not explored.

### Implications for practice

This commentary is a timely contribution to the increasingly important role played by RGT in paediatric care. RGT has become a vital tool in paediatric practice, particularly for critically ill children with suspected genetic diseases. It provides a definitive diagnosis in a significant number of cases, leading to crucial changes in clinical management, such as initiating targeted treatments or avoiding invasive procedures (Bick et al. 2019; McDermott et al. 2022). The 2022 economic evaluation by Goranitis et al. (Goranitis et al. 2022) was important in confirming that faster, earlier testing is cost-effective, primarily by reducing lengthy and expensive “diagnostic odysseys”. Since 2022, the field has advanced significantly beyond the scope of the Goranitis et al. study. Key recent developments include:

- **Mainstreaming as standard of care:** A major policy shift has seen national health systems formally adopt RGT as a standard of care. In the UK, the NHS has transitioned to using rapid whole-genome sequencing for all eligible children in intensive care (NHS England 2022). Similarly, the Human Genetics Society of Australasia (HGSA) now recommends rapid whole-genome sequencing as the standard of care in acute paediatric settings, favouring its superior diagnostic scope over exome sequencing (Vears et al. 2024).
- **Expansion to Newborn Screening:** The paradigm has expanded from the reactive diagnosis of sick infants to the proactive screening of healthy newborns. Major research initiatives, like the UK’s Generation Study, are now underway to evaluate using whole-genome sequencing to screen up to 100,000 babies for hundreds of treatable childhood-onset conditions, aiming to generate evidence for future national screening policy (NHS England 2022).

This rapid progress has exposed new challenges not fully addressed in earlier work. A critical implementation gap has emerged between the availability of the technology and the genomic literacy of the frontline paediatric workforce, creating a bottleneck to effective care (Kansal 2025). Furthermore, the psychosocial impact on families is now understood to be more complex, with research exploring the value families place on different outcomes beyond a simple diagnosis (Goranitis et al. 2021). Finally, the expansion into population-level newborn screening raises profound new ethical questions regarding the handling of predictive information, data stewardship, and a child’s right to an open future, shifting the ethical concerns from immediate clinical benefit to a more complex balance of future risks and benefits (Gyngell et al. 2019).

These recent developments should be acknowledged to gain a fuller picture of the barriers and facilitators to the wider implementation of RGT, beyond what was outlined by Goranitis et al. (Goranitis et al. 2022). In a recent article, Kansal (Kansal 2025) importantly highlights key actions to achieve wider RGT implementation, such as increasing accessibility to RGT, educating healthcare professionals and families, timely responding to changes in RGT costs, examining the differential cost-effectiveness for different paediatric disorders, and providing evidence-based clinical guidelines.

As Goranitis et al. recognise, beyond the clinical and cost-effectiveness of ultra-RGT, its wider implementation requires systemic coordination and communication across the different pathway components (laboratory, paediatric wards, critical care units, primary, and other community care services) in addition to investments in education and training of the providers involved (Goranitis et al. 2022). With early ultra-RGT the timeframe of the test initiation and delivery of subsequent results is reduced, making laboratory capacity a potential challenge (Stark and Ellard 2022). However, where the accuracy of the test results gains consistency, the quicker turnaround will likely increase the buy-in from clinicians and families of affected children (Best et al. 2020).

Other potential barriers are the unit costs of RGT (AU$ 3,960) and ultra-RGT (AU$ 12,000) which were quite higher than that of SGT (AU$ 2,100) (Goranitis et al. 2022). Here economies of scale could be reaped through wide-scale implementation of rapid or ultra-RGT strategies to help reduce the related fixed costs in the long-term, as investment costs to expand tests’ capacity are absorbed (Stark and Ellard 2022).

## Conclusions

In this commentary we expanded upon the limitations of Goranitis et al. (Goranitis et al. 2022) to take into account recent developments key important factors not addressed by the study which would contribute to a wider use of RGT for critically ill infants and children.

Future studies based on more sites, larger sample sizes and longer-term analysis will help strengthen the characterisation of the patients’ variability and heterogeneity (e.g. more robust sub-group analyses by condition severity) while investigating the cost-effectiveness of the RGT strategies at longer timeframes. Sub-group analyses investigating the differential cost-effectiveness of RGT according to different clinical disorders and severity levels are also needed to aid prioritisation decisions. Changes in costs associated with the RGT strategies will also likely impact on the outlook on the relative cost-effectiveness and the consequences on wider implementation (Vears et al. 2024).

All in all, research in genomic medicine can provide equitable access to improved prevention, diagnosis and treatment of disease yielding major benefits to the children’s future health (NHS England 2022). With this commentary we emphasised the multifactorial and wide-ranging aspects associated with RGT which will likely impact on its future wide-scale implementation. Our commentary helps summarizing and validating important evidence that can help communicating the value (for money) of RGT to healthcare professionals and families. Future research in RGT will be key in ensuring that vital health gains in infants and children are not foregone.

## Reflective questions

1) What are the main economic advantages and disadvantages of using rapid genomic testing for critically ill children?
2) What do you think are the main strengths and limitations in the economic evaluation by Goranitis et al. (2022)?
3) Which other barriers and facilitators (beyond those pointed out by the authors of the commentary) do you suggest may have an impact on a wider implementation of rapid genomic testing?

## Key points

- Rapid genomic testing could be clinically effective and cost-saving when used as a diagnostic strategy in the care of critically ill children.
- This commentary critically appraised an existing economic evaluation of rapid genomic testing and ultra-rapid genomic testing, highlighting limitations with its methods and reporting of results.
- We also built upon its findings by drawing the organisational and economic implications of a wide-scale implementation of rapid genomic testing.
